# A potential preventive method for scar stenosis after esophageal endoscopic mucosal resection using human amniotic epithelial cells in a porcine model

**DOI:** 10.1002/deo2.104

**Published:** 2022-03-09

**Authors:** Yuji Konno, Chiaki Sato, Kazuaki Tokodai, Masatoshi Saito, Tetsuro Hoshiai, Yusuke Taniyama, Hiroshi Okamoto, Toshiaki Fukutomi, Yohei Ozawa, Naoto Ujiie, Ken Koseki, Ryohei Ando, Kozue Takahashi, Yasuharu Shinozaki, Michiaki Unno, Takashi Kamei

**Affiliations:** ^1^ Department of Surgery Tohoku University Graduate School of Medicine Miyagi Japan; ^2^ Department of Maternal and Fetal Therapeutics Tohoku University Graduate School of Medicine Miyagi Japan; ^3^ Department of Gynecology and Obstetrics Tohoku University Graduate School of Medicine Miyagi Japan

**Keywords:** cell transplantation, endoscopic submucosal dissection, esophageal stenosis, regenerative medicine

## Abstract

**Objectives:**

The current methods employed for esophageal endoscopic mucosal resection (EMR) involve the risk of adverse postprocedural complications. Therefore, this study aimed to develop a new method to prevent stenosis following a resection procedure using human amniotic epithelial cells in a porcine model.

**Methods:**

With the consent of a woman who underwent a cesarean section, amniotic epithelial cells were isolated from the amniotic membrane of the delivered placenta. Six swine were used for this study. Under general anesthesia, four EMRs using cap‐fitted microscope ulcers were performed on each porcine esophagus. Of the four ulcers, the two on the oral side were treated by injecting human amniotic epithelial (AE group) cells, and the remaining two on the anal side were left untreated (control group). One week after the procedure, the swine were sacrificed, and the ulcers were evaluated. The epithelialization rate was calculated by dividing the length of the epithelialized portion of each section by the length of the ulcer, which was determined using an optical microscope. Moreover, the mucosal thickening in each section was measured in terms of diameter.

**Results:**

The epithelialization rate was significantly higher in the AE group than in the control group. Mucosal thickening was not significantly different between the groups.

**Conclusions:**

Transplanting amniotic epithelial cells into the ulcer promoted ulcer epithelialization. Amniotic epithelial cell transplantation is a potential method for the management of ulcer scar stenosis following esophageal endoscopic submucosal dissection.

## BACKGROUND

Endoscopic submucosal dissection (ESD)^1^ enables early batch resection of malignant tumors of the gastrointestinal tract. It is widely used because of its high curability and minimal invasiveness.^2^

Postoperative ulcer scar stenosis is a major complication of ESD. Endoscopic balloon dilation (EBD), local steroid therapy, oral steroid therapy, and other stenosis prevention methods have been developed to treat this complication. EBD is one of the leading treatments for benign esophageal stenosis^3^; however, it can cause serious adverse events, including perforation and bleeding.^4^ Steroid treatment prevents stenosis by suppressing inflammation and fibrosis, with steroid local injection therapy emerging as one of the most popular treatments for preventing post‐esophageal ESD stenosis. However, in studies examining local steroid therapy, the use of steroids has been reported to weaken the esophageal wall and cause perforation.^5^ In addition, adverse events including secondary adrenal gland dysfunction, diabetes, and infectious diseases due to oral steroid administration have become problematic.^6^

An approach that is free of these adverse events is regenerative medicine to prevent stenosis. Human embryonic stem (ES) cells^7^ are an established cell source used in regenerative medicine as they are pluripotent and can differentiate into any specialized cell type. However, ES cells are harvested from fertilized human eggs and are, therefore, subject to ethical problems.

Sakurai et al.^8^ noted that epithelialization of an ulcer surface was significantly promoted when epithelial keratinocytes isolated from human oral mucosal tissue were locally injected into the submucosa of a porcine esophagus immediately after endoscopic mucosal resection (EMR). Ohki et al.^9^, ^10^ specified a method of culturing oral mucosal epithelial cells, preparing a cell sheet, and transplanting it after esophageal ESD surgery. Although this technique can prevent stenosis and has been applied clinically, it has not been widely used because processes such as culturing are complicated, and their overall cost is high.

Therefore, we focused on the amniotic membrane. Amniotic membrane epithelial cells are derived from the upper blastoderm layer that forms the embryo, which is composed of amniotic membrane epithelial cells, basement membrane, and a stromal layer. Since the upper blastoderm can be used as a reference in differentiating the endoderm, mesoderm, and ectoderm, it has long been hypothesized that amniotic epithelial (AE) cells have stem cell‐like properties.^11^, ^12^ Several analyses on the pluripotency of AE cells have been performed, and differentiation into hepatocyte‐like cells^11^, ^13^ and insulin‐producing islet cell‐like cells^14^ have been reported. AE cells promote ulcer healing by producing cytokines.^15^ In the field of ophthalmology, amniotic membrane transplantation has been performed for intractable ocular surface diseases and found to be highly effective.^16^

We hypothesized that AE cells result in the early healing of esophageal EMR/ESD ulcers. Therefore, we believe that the transplantation of amniotic membrane cells into esophageal ulcers could be a new preventive method for ulcer scarring. We verified the efficacy of this method by experimentally creating an endoscopically treated ulcer in the porcine esophagus and transplanting AE cells into it.

## METHODS

### Amniotic membrane collection, AE cell separation, and thawing

This study was approved by the Ethics Committee of the Graduate School of Medicine, Tohoku University (Permission number: 2019‐1‐430). The amniotic membrane was collected with the consent of a pregnant woman undergoing a scheduled cesarean section at the Department of Obstetrics, Tohoku University Hospital. The amniotic membrane was collected from the placenta, which was removed after delivery by cesarean section. The isolation of AE cells was performed according to the protocol of Gramignoli et al.^17^ TrypLE Select Enzyme (10X) (Thermo Fisher Scientific, Waltham, MA, USA) was added to 1 g of amniotic membrane and incubated at 35 rpm for 30 min at 37°C to isolate AE cells. The AE cells that had undergone the filtration process were placed in the cell preservation solution NutriFreez D10 (Biological Industries, Cromwell, CT, USA) and stored frozen at –80°C or lower.

Cryopreserved AE cells were thawed at 37°C for 1 min and washed with Dulbecco's phosphate‐buffered saline (PBS). Two milliliters of normal saline were then added per 1.0 × 10⁷ of AE cells.

### Fluorescent labeling of AE cells

CellTracker (Thermo Fisher Scientific) CM‐DiI was used as the fluorescent label. The adjustment of CM‐DiI was performed according to the protocol of the package insert and the reference of Fang et al.^18^ The AE cell suspension was prepared by adding PBS to 1.0 × 10^6^ cells/ml, and CM‐DiI was added to a final concentration of 5 μM. This was then incubated at 37°C for 5 min and then at 4°C for 20 min for labeling. After labeling, the cells were washed with PBS and suspended in 2 ml of normal saline per 1.0 × 10^7^ AE cells for use.

### Transplantation of AE cells into porcine esophageal ulcers

Six swine were used for this study. Under general anesthesia, four EMRs using cap‐fitted microscope (EMRC) ulcers were created in each porcine esophagus. The EMRC method was as follows. Saline was locally injected into the submucosa and bulged. A tip hood (cap) (Distal Attachment, MAJ‐295; Olympus Medical Systems Corp., Tokyo, Japan) was attached to the gastrointestinal endoscope. We created a loop with a high‐frequency snare so that it could be hooked on the claw of the tip hood. The mucosa was aspirated using a gastrointestinal endoscope, then the snare was squeezed. The generator used was the VIO300D (ERBE Elektromedizin GmbH, Tübingen, Germany), and the settings were: ENDO CUT effect of 2, FORCED COAG effect of 3, and a maximum wattage of 60. The strangled snare was appropriately coagulated and incised, then mucosa was excised to create an ulcer.

**FIGURE 1:**
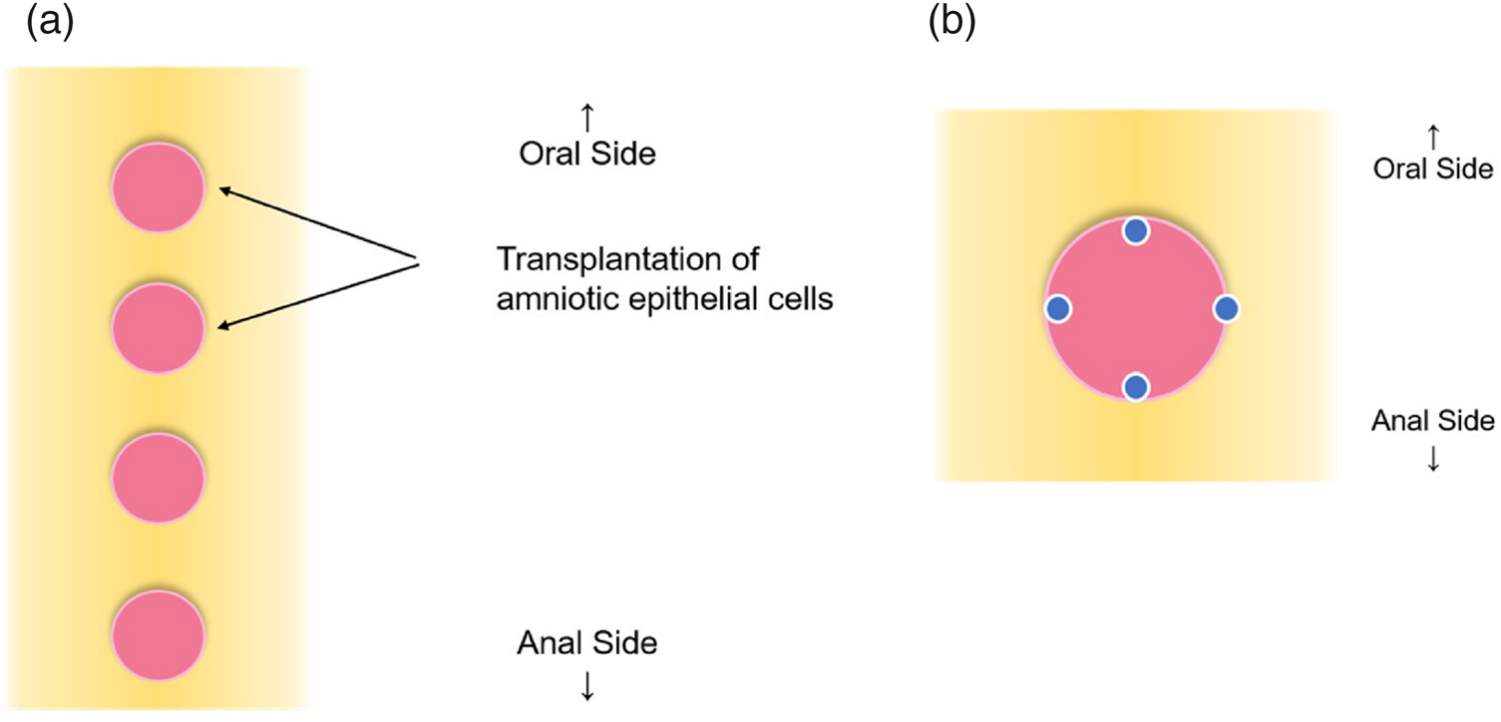
Schematic diagram of EMRC ulcer. (a) Multiple EMRC ulcers were created in one pig. This figure shows a schematic diagram in which four ulcers were created. Of these, amniotic epithelial cells were injected locally into the two oral‐side ulcers. (b) Shamer‐like amniotic epithelial cells were locally injected over four margins of the ulcer. A 2 ml suspension containing 1.0 × 10^7^ amniotic epithelial cells per ulcer was locally injected. EMRC, endoscopic mucosal resection using cap‐fitted microscope

Of the four ulcers, the two on the oral side were treated with human AE cell transplantation (AE group), and the remaining two on the anal side were left untreated (control group) (Figure 1a). For AE group ulcers, a suspension of 1.0 × 10^7^ AE cells, in a volume of 2 ml, was locally injected into the ulcer; 0.5 ml was locally injected into each of the four points on the ulcer margin, as shown in Figure 1b.

Only drinking water was given on the first day of the operation. Feeding was resumed 1 day after the operation. Famotidine 40 mg/day was administered up to 3 days after surgery and acetaminophen (20% fine granules) 800 mg/day was administered for 1 week after surgery for analgesic purposes.

One week later, general anesthesia was administered, and each ulcer was observed via gastrointestinal endoscopy. After observation, under deep anesthesia, a 20% potassium chloride solution was intravenously administered to induce death and the esophagus was removed. There are two reasons for assessing epithelialization 1 week after surgery. First, existing studies have reported mild to severe stenosis 14 days after ESD.^19^, ^20^ Euthanasia was considered when the oral intake of the pigs deteriorated significantly, but the experiment could be carried out as scheduled within 1 week after the operation so we could detect signs of slight stenosis. Second, Ota et al. stated that ulcer healing after esophageal EMR/ESD is as short as 21 days.^21^ When the ulcer was completely healed, it would be difficult to compare epithelialization, so we decided to observe it 1 week later.

### Pathologic evaluation

**FIGURE 2:**
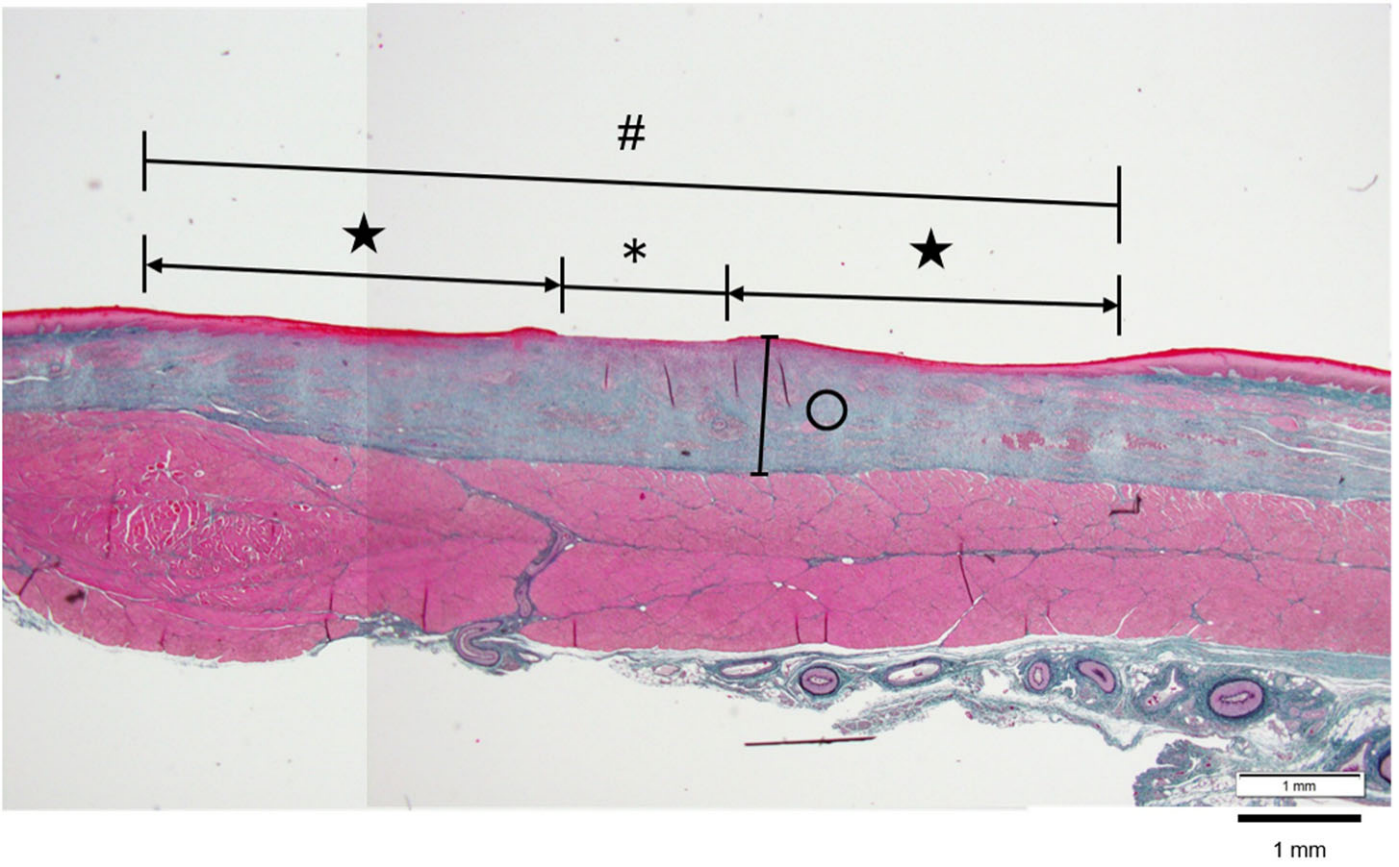
Histological findings one week after EMRC (Elastica Masson staining). #: Long diameter of ulcer, ★: Epithelialized part, *: Non‐epithelialized part, and ○: Maximum mucosal thickening diameter of ulcer. The length of the ulcer and the length of the epithelialized part were measured in each section. The epithelialization rate for each ulcer was next determined. In addition, the maximum diameter of mucosal thickening was measured for each section. EMRC, endoscopic mucosal resection using cap‐fitted microscope

Each excised specimen was fixed using 10% phosphate‐buffered formalin. After embedding in paraffin, the samples were sliced and hematoxylin and eosin staining, as well as Elastica Masson staining, was performed. Each ulcer was evaluated using an optical microscope and the imaging software cellSens Standard (Olympus Corp.). An ulcer was defined as the part where the mucosa was thinned, the muscularis mucosae were torn, and the epithelium was defective. Epithelialization was defined as the area where the stratified squamous epithelium was regenerated and keratinized within the ulcer. The length of the ulcer in each section and the length of the epithelialized part were measured with an optical microscope. The length of the epithelialized part of each section, divided by the length of the ulcer, was defined as the epithelialization rate. To evaluate the thickening of the mucosa due to fibrosis caused by ulcer scarring, the maximum diameter from the upper end of the muscularis propria to the upper end of the mucosa in each section was measured as the mucosal thickening diameter (Figure 2). In addition, a fluorescence microscope was used to assess unstained slides to observe the dynamics of the labeled AE cells.

### Statistical analysis

The statistical software JMP Pro 15.0.0 (SAS Institute, Cary, NC, USA) was used for statistical analysis. The median ulcer length, epithelialized length, epithelialization rate, and mucosal thickening diameter were compared between the AE and control groups. Comparisons were performed using the Wilcoxon test, and statistical significance was set at *p* < 0.05.

## RESULTS

### EMRC ulcer creation with gastrointestinal endoscopy

**FIGURE 3:**
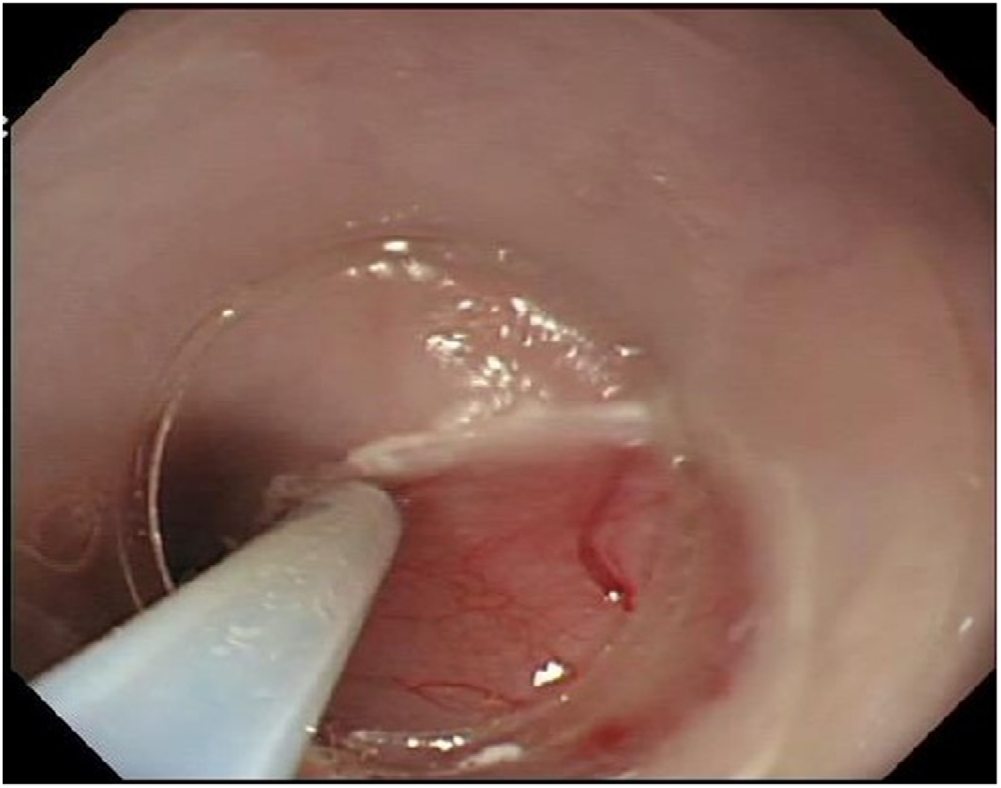
Amniotic epithelial cell transplantation. An endoscopic image of amniotic epithelial cell transplantation. The amniotic epithelial cell suspension was locally injected into the EMRC ulcer. EMRC, endoscopic mucosal resection using cap‐fitted microscope

Twenty‐four EMRC ulcers were created in the six pigs. Two EMRC ulcers were fused in one of the pigs, resulting in 22 experimental EMRC ulcers. An AE cell suspension was locally injected into the ulcers of the AE group (Figure 3). For pigs with healed ulcers, one of the two remaining effective ulcers (the oral side) was used as the AE group. The purity of the isolated human AE cells was 100% and the median viability was 80.50% (interquartile range [IQR]: 79.2%–82.5%). There were no adverse events, such as perforation, during the creation of the ulcers.

The pigs survived for 1 week after the surgery. During this course, there were no other serious adverse events, and the general condition of the specimens was good as they had been treated as safely as possible.

### Observation of ulcers at 1 week using gastrointestinal endoscopy

**FIGURE 4:**
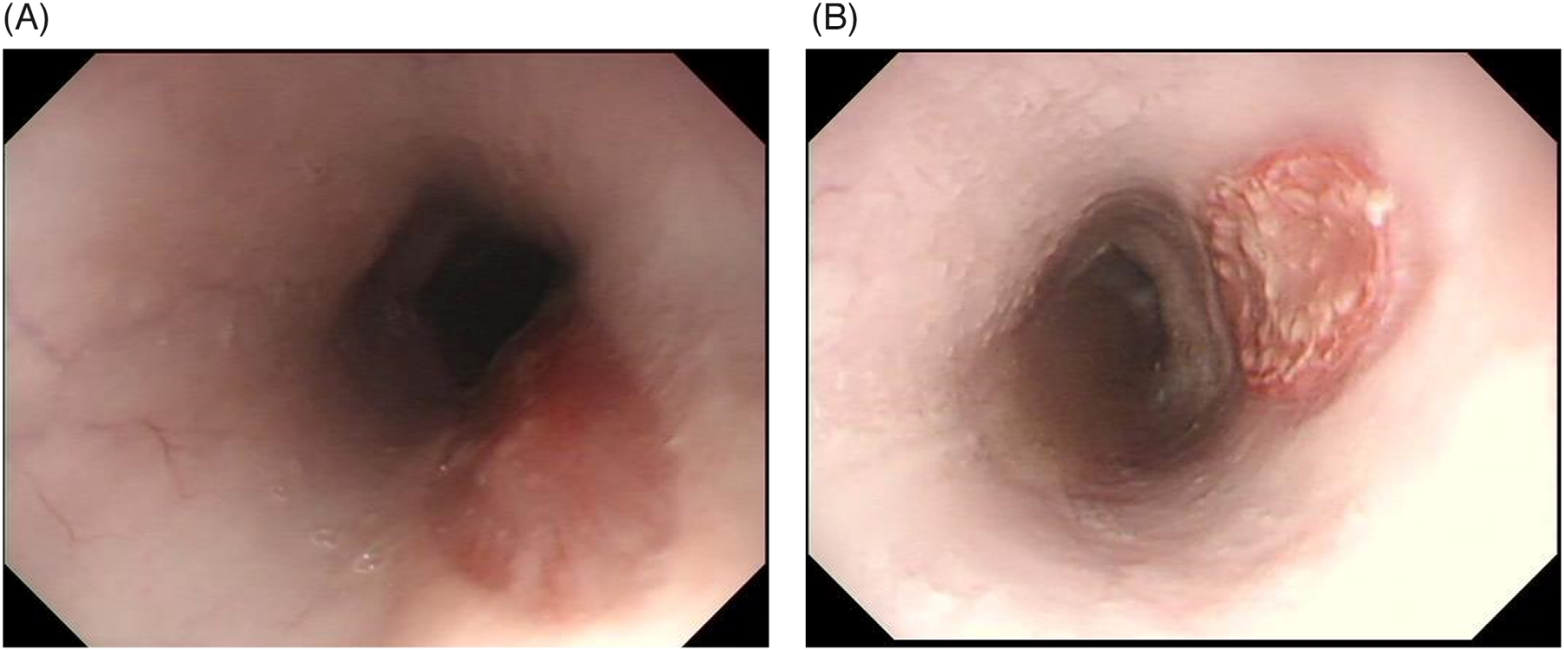
Endoscopic image of ulcer 1 week after EMRC. (a) AE group. (b) Control group. The ulcer tended to heal immediately after the ulcer was created. No findings, such as perforations, were observed. AE, amniotic epithelial cells

One week later, when the esophagus of each pig was observed with a gastrointestinal endoscope under general anesthesia, no stenosis had occurred in any esophagus, with ulcers tending to heal in both the AE and control groups (Figure 4).

### Observation of excised specimens

**FIGURE 5:**
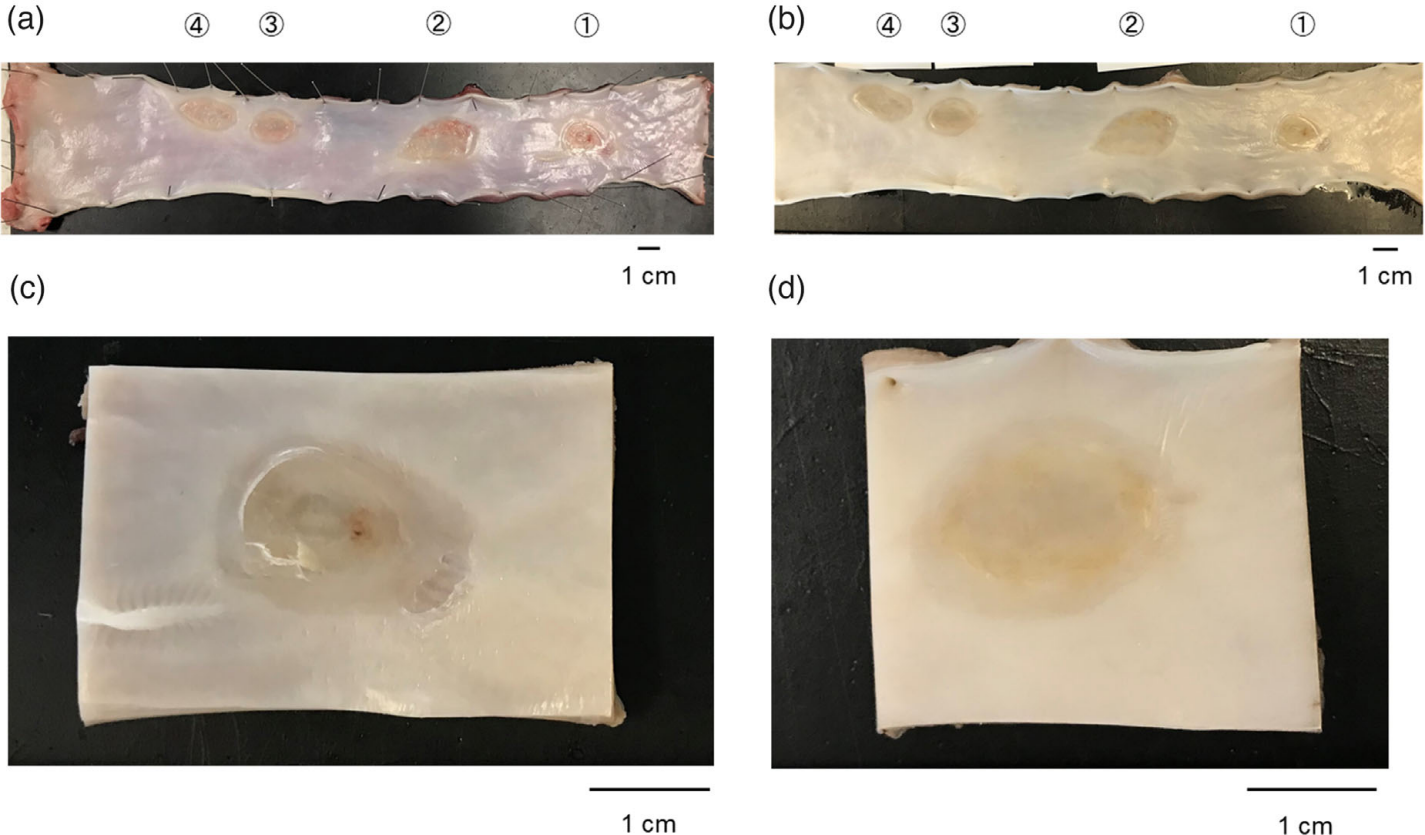
Post‐EMRC ulcer specimen photo. (a) Before specimen fixation. (b) After fixing the specimen. (c) AE group ulcer after specimen fixation (ulcer No.1). (d) Control group ulcer after specimen fixation (ulcer No. 3). In the porcine esophageal specimen in the photograph, the right side is the oral side, the left side is the anal side. Ulcers Nos.1 and 2 are the AE group and ulcers Nos. 3 and 4 are the control group. Macroscopically, there was no significant change in ulcer healing. EMRC, endoscopic mucosal resection using cap‐fitted microscope; AE, amniotic epithelial cells

No significant macroscopic changes were observed in the ulcers of either the AE group or the control group (Figure 5).

**FIGURE 6:**
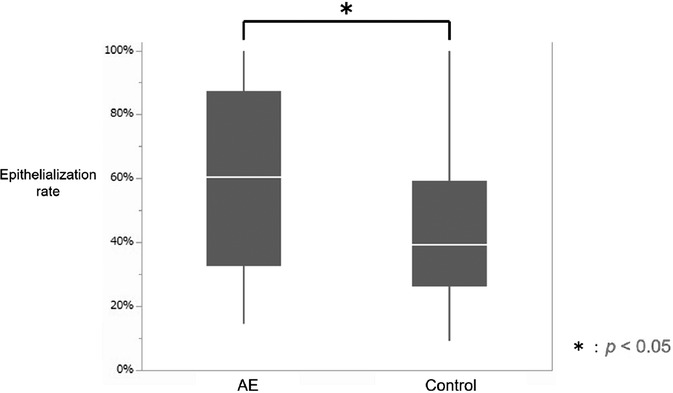
Comparison of epithelialization rates between two groups. A graph comparing the epithelialization rates in the AE and control groups. The epithelialization rate was higher in the AE group, with a statistically significant difference (* *p* < 0.05) observed. AE, amniotic epithelial cells

The total number of ulcers created by EMRC was 22, comprising 11 ulcers each in the AE and control groups. Pathologically confirmed sections included 83 and 80 sections in the AE and control groups, respectively. The median ulcer major axis measured 24 mm (21–32 mm) in the AE group and 27 mm (22.5–31.8 mm) in the control group; there was no significant difference between the two groups (*p* = 0.6355). The median EMRC specimen on the major axis measured 21 mm (16–25 mm) in the AE group and 22 mm (12–31 mm) in the control group, with no significant difference between the two groups (*p* = 0.9738). The AE group had a median ulcer length of 9432.9 μm (4914.1–13695.4 μm) and the epithelialized area had a median length of 5533.6 μm (3900.5–7527.8 μm). In the control group, the median ulcer length of 9411.7 μm. (4734.4–13345.1 μm) and the median length of the epithelialized part was 4425.4 μm (2867.5–5368.1 μm). The epithelialization rate was obtained by dividing the length of the epithelialized portion of each section by the length of the ulcer, with the exception of one ulcer in the AE group and one ulcer in the control group, in which the epithelialization rate of each section was 100%. The final evaluation included 58 sections of 10 ulcers in the AE group and 55 sections of 10 ulcers in the control group, excluding one section at each end of each ulcer. The median epithelialization rate in the AE group was 60.4% (32.7%–87.3%) and the median in the control group was 39.1% (26.0%–59.2%). The epithelialization rate was significantly higher in the AE group (*p* = 0.0178) (Figure 6).

**FIGURE 7:**
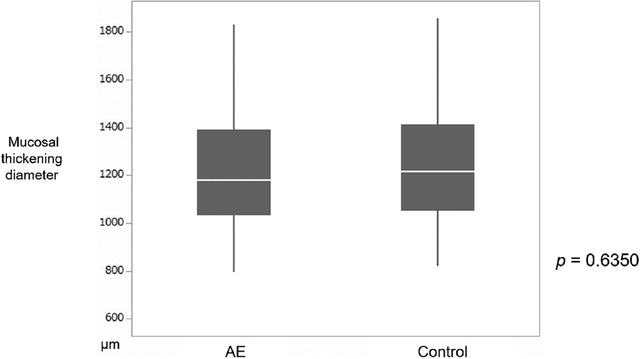
Comparison between two groups regarding mucosal thickening diameter. Graph of mucosal thickening diameter comparing the AE and control groups. No significant differences were observed between the two groups. AE, amniotic epithelial cells

The median mucosal thickening diameter was 1169.6 μm (1009.2–1455.9 μm) in the AE group and 1214.7 μm (1049.6–1414.7 μm) in the control group; no significant difference was observed (*p* = 0.6350) (Figure 7).

### Observation of fluorescently labeled AE cells

**FIGURE 8:**
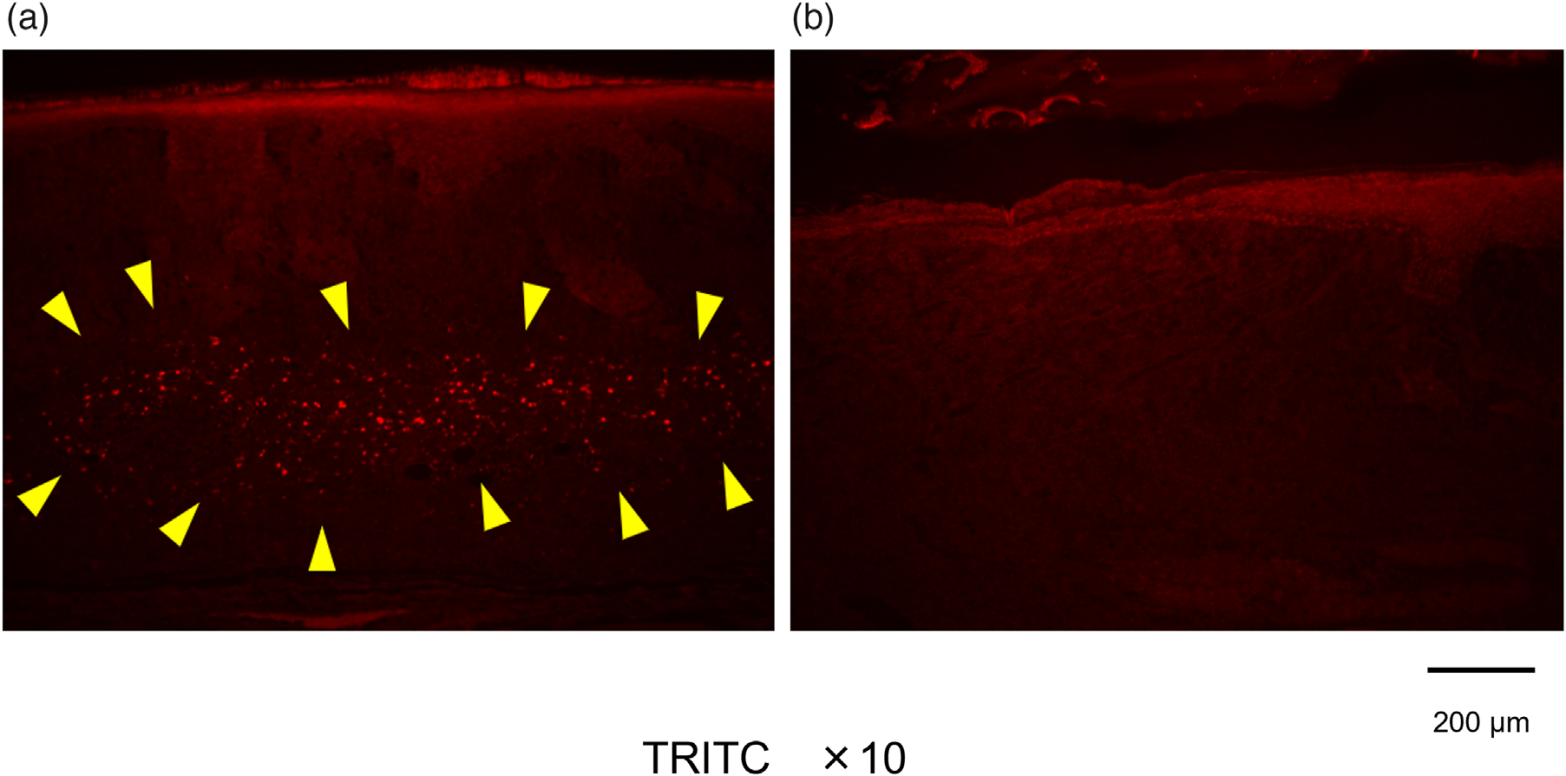
Fluorescence microscope findings. (a) AE group (TRITC x 10 Exposure time 0.5 s). (b) Control group (TRITC x 10 Exposure time 0.5 s). Fluorescent label‐positive cell clusters were observed in the sections of the AE group, as shown in Figure (a) (arrowheads). No fluorescent label‐positive cell clusters were observed in the control group. AE, amniotic epithelial cells; TRITC, tetramethylrhodamine isothiocyanate

**FIGURE 9:**
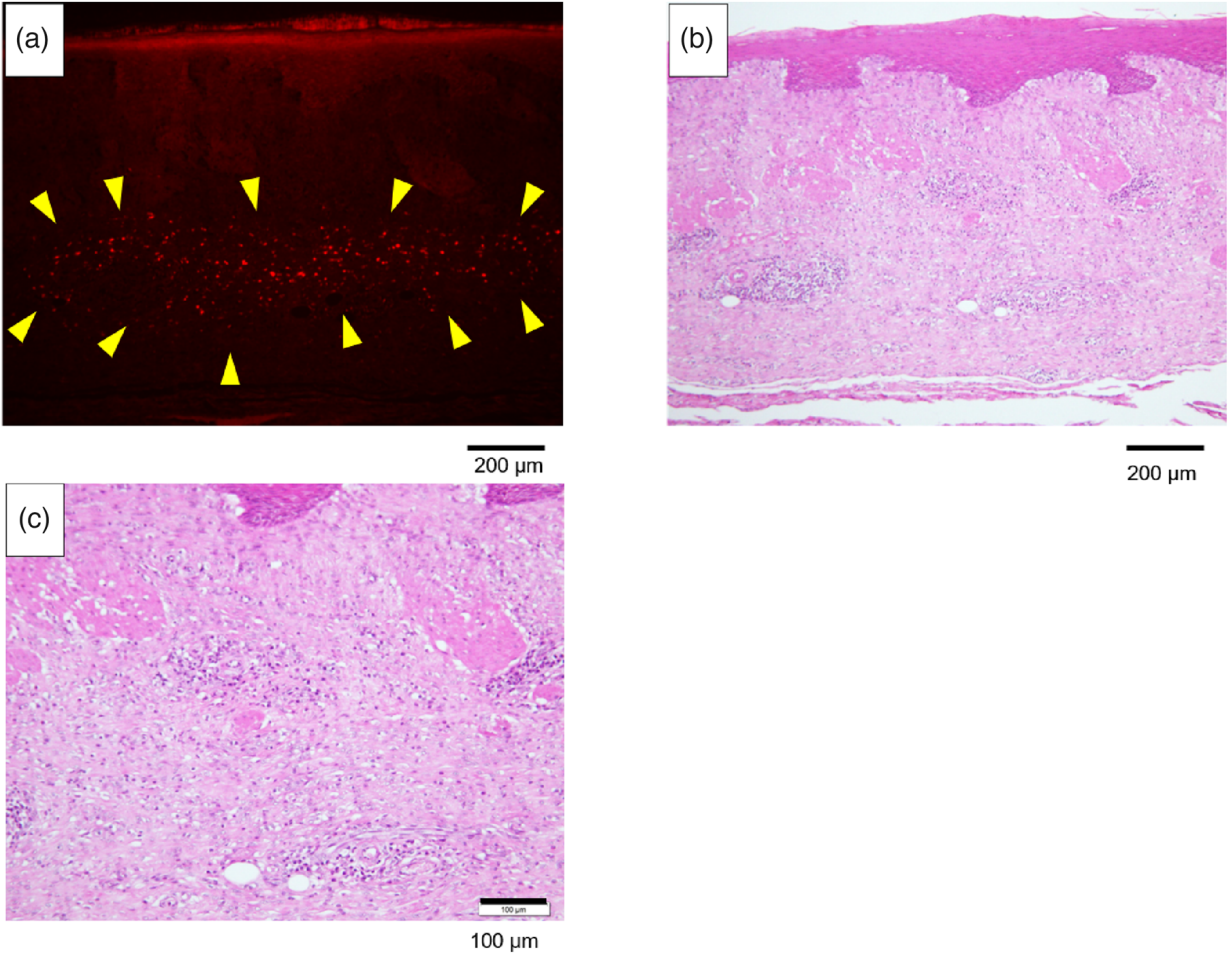
Fluorescent label‐positive cells in the AE group. (a) Fluorescence microscope findings (TRITC x 10 Exposure time 0.5 s). (b) Hematoxylin/eosin staining 10x. (c) Hematoxylin/eosin staining 20x. The fluorescent labeled‐positive cell cluster was localized within the submucosa. Figures (b) and (c) show hematoxylin and eosin staining at the same site; no significant infiltration of histiocytes was observed in the surrounding areas. No significant infiltration of histiocytes was observed in the control group either. AE, amniotic epithelial cells; TRITC, tetramethylrhodamine isothiocyanate

A collection of cells believed to be fluorescently labeled AE cells was observed in the preparation of the AE group whereas no cell aggregation was observed in the control group (Figure 8). It could not be confirmed if these cells had differentiated into esophageal mucosal epithelial cells and/or other cells (Figure 9).

## DISCUSSION

We conducted the first study to use AE cells isolated from the human amniotic membrane in the prevention of post‐esophageal ESD ulcer scar stenosis. The results of this study suggest that local injection of AE cells into ESD ulcers promotes the epithelialization of ulcers and increases the rate of epithelialization. In addition, fluorescently labeled AE cells were found to be localized within the locally injected submucosa of the esophagus.

Amniotic membrane cells are known to express growth factors that promote wound healing. Koizumi et al.^22^ speculated that growth factors, such as epidermal growth factor and keratinocyte growth factor, expressed in amniotic cells contribute to shortening the epithelial repair period. In addition, Zhao et al. found that AE cells also play a role in regulating cell proliferation and promoting the induction and differentiation of keratinocytes through the mitogen‐activated protein kinase pathway and phosphoinositide 3‐kinase/Akt pathway. When a mouse model of a wound with a skin defect was treated by injecting a medium containing AE cells, the healing of the wound was significantly improved as compared with controls.^15^ In this study, it is possible that the activation of growth factors and cell differentiation promoting wound healing contributed to the promotion of epithelialization, leading to an improvement in ulcer healing in the AE group. It was not clear whether the AE cells differentiated into esophageal mucosal epithelial cells under observation using a fluorescence microscope; however, the transplanted AE cells remained in the locally injected part even after 1 week. This is the first study in which we observed the dynamics of amniotic cells transplanted into ulcers after esophageal endoscopy using techniques described in previous studies.

We found no significant difference in the mucosal thickening diameter between the AE and control groups. Liu et al.^19^ cited mucosal deficiency as one contributing factor in scar stenosis of post‐esophageal ESD ulcers and stated that the larger the mucosal deficiency, the more likely it was to become stenotic. Unlike peripheral ESD ulcers, the EMRC ulcer created in this study was a local ulcer with a partial mucosal defect. We hypothesize that this may be the reason why there was no significant difference in the diameter of mucosal thickening. Scar stenosis in post‐ESD ulcers is thought to be due in part to the persistence of local chronic inflammation, healing, and the development of hypertrophic scars on the skin.^8^, ^23^ Hermans^23^ stated that rapid re‐epithelialization was an important factor in preventing hypertrophic scars. Even in post‐esophageal ESD ulcer scars, scar stenosis may be reduced if re‐epithelialization is accelerated by promoting healing. It has also been reported that amniotic membrane cells have anti‐inflammatory effects. Miyamoto et al. administered a solution in which amniotic cells were suspended by enema to rats with induced colitis, which improved intestinal inflammation, levels of infiltrated neutrophils and monocytes in the mucosa, and expressed inflammatory cytokines.^24^ It is believed that the anti‐inflammatory effect of the amniotic membrane prevents chronic local inflammation, which may prevent ulcer scar stenosis.

There are several limitations to this study. First of all, this study was done with EMRC rather than ESD. In the future, we would like to continue research to see if there is a similar effect on circumferential ESD ulcers. Second, it is possible that AE cells have anti‐inflammatory effects, and the expression of growth factors contributes to the promotion of epithelialization. However, this study did not evaluate the number of inflammatory cells or the quantification of the growth factors. It is necessary to clarify these in the future. Pre‐stage experiments, including animal experiments as well as other studies, should also be repeated with the goal of transferring findings into clinical applications.

In conclusion, to develop a method for preventing esophageal ESD post‐ulcer scar stenosis, AE cells were transplanted and evaluated in animal experiments. Transplantation of AE cells significantly promoted EMRC ulcer epithelialization. This may have contributed to the promotion of epithelialization through the activation of growth factors and the promotion of cell differentiation by AE cells. This study suggests that AE cell transplantation is a potential method for preventing post‐ESD ulcer scar stenosis.

## CONFLICT OF INTEREST

The authors declare no conflict of interest.

## FUNDING INFORMATION

This research received no specific grant from any funding agency in the public, commercial, or not‐for‐profit sectors.
